# Degradation of Extracellular NAD^+^ Intermediates in Cultures of Human HEK293 Cells

**DOI:** 10.3390/metabo9120293

**Published:** 2019-11-29

**Authors:** Veronika Kulikova, Konstantin Shabalin, Kirill Nerinovski, Alexander Yakimov, Maria Svetlova, Ljudmila Solovjeva, Andrey Kropotov, Mikhail Khodorkovskiy, Marie E. Migaud, Mathias Ziegler, Andrey Nikiforov

**Affiliations:** 1 Institute of Cytology, Russian Academy of Sciences, St. Petersburg 194064, Russia; veronika.a.kulikova@gmail.com (V.K.); svetlma@mail.ru (M.S.); mila.solovjeva@gmail.com (L.S.); a.kropotov@gmail.com (A.K.); 2 Peter the Great St. Petersburg Polytechnic University, St. Petersburg 195251, Russia; yaleks@gmail.com (A.Y.); khodorkovskii@gmail.com (M.K.); 3 Sechenov Institute of Evolutionary Physiology and Biochemistry, Russian Academy of Sciences, St. Petersburg 194223, Russia; 4 Petersburg Nuclear Physics Institute Named by B.P. Konstantinov of National Research Centre “Kurchatov Institute”, Gatchina 188300, Russia; konstantin.shabalin@gmail.com; 5 Department of Nuclear Physics Research Methods, St. Petersburg State University, St. Petersburg 199034, Russia; nerinovski@yandex.ru; 6 Mitchell Cancer Institute, University of South Alabama, Mobile, AL 36604, USA; mmigaud@health.southalabama.edu; 7 Department of Biomedicine, University of Bergen, 5020 Bergen, Norway; Mathias.Ziegler@uib.no

**Keywords:** NAD metabolism, extracellular NAD^+^ intermediates, NMR spectroscopy, human cells

## Abstract

Nicotinamide adenine dinucleotide (NAD) is an essential redox carrier, whereas its degradation is a key element of important signaling pathways. Human cells replenish their NAD contents through NAD biosynthesis from extracellular precursors. These precursors encompass bases nicotinamide (Nam) and nicotinic acid and their corresponding nucleosides nicotinamide riboside (NR) and nicotinic acid riboside (NAR), now collectively referred to as vitamin B3. In addition, extracellular NAD^+^ and nicotinamide mononucleotide (NMN), and potentially their deamidated counterparts, nicotinic acid adenine dinucleotide (NAAD) and nicotinic acid mononucleotide (NAMN), may serve as precursors of intracellular NAD. However, it is still debated whether nucleotides enter cells directly or whether they are converted to nucleosides and bases prior to uptake into cells. Here, we studied the metabolism of extracellular NAD^+^ and its derivatives in human HEK293 cells using normal and serum-free culture medium. Using medium containing 10% fetal bovine serum (FBS), mono- and dinucleotides were degraded to the corresponding nucleosides. In turn, the nucleosides were cleaved to their corresponding bases. Degradation was also observed in culture medium alone, in the absence of cells, indicating that FBS contains enzymatic activities which degrade NAD^+^ intermediates. Surprisingly, NR was also rather efficiently hydrolyzed to Nam in the absence of FBS. When cultivated in serum-free medium, HEK293 cells efficiently cleaved NAD^+^ and NAAD to NMN and NAMN. NMN exhibited rather high stability in cell culture, but was partially metabolized to NR. Using pharmacological inhibitors of plasma membrane transporters, we also showed that extracellular cleavage of NAD^+^ and NMN to NR is a prerequisite for using these nucleotides to maintain intracellular NAD contents. We also present evidence that, besides spontaneous hydrolysis, NR is intensively metabolized in cell culture by intracellular conversion to Nam. Our results demonstrate that both the cultured cells and the culture medium mediate a rather active conversion of NAD^+^ intermediates. Consequently, in studies of precursor supplementation and uptake, the culture conditions need to be carefully defined.

## 1. Introduction

Nicotinamide adenine dinucleotide (NAD) is the coenzyme of dehydrogenases that catalyze the redox reactions in central metabolic pathways. In addition, the degradation of NAD is a key element of a multitude of signaling events in which this dinucleotide is used for the modification of proteins (deacylation and ADP-ribosylation), but also as a precursor of signaling molecules. In recent years, it has become increasingly clear that NAD-dependent regulatory pathways are present in all organisms. These pathways do not merely mediate the adaptation of metabolism to changes of the environment, but also participate in the control of fundamental cellular processes including aging and the regulation of the cell cycle as well as gene expression, DNA repair, cell differentiation, calcium signaling, and apoptosis [1,2,3].

**Figure 1:**
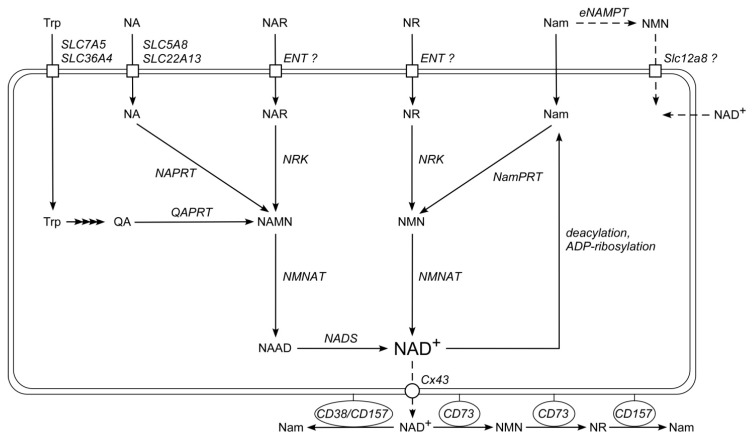
Nicotinamide adenine dinucleotide (NAD^+^) biosynthesis in human cells. Tryptophan (Trp), pyridinic bases nicotinamide (Nam) and nicotinic acid (NA), and the nucleosides nicotinamide riboside (NR) and nicotinic acid riboside (NAR) are precursors for intracellular NAD^+^ synthesis. Tryptophan is converted to quinolinic acid (QA) in a series of enzymatic reactions; nicotinic acid mononucleotide (NAMN) is synthesized from QA by quinolinate phosphoribosyltransferase (QAPRT). Nicotinamide phosphoribosyltransferase (NamPRT) converts Nam into nicotinamide mononucleotide (NMN), which, in turn, is adenylated to NAD^+^ by nicotinamide mononucleotide adenylyltransferase (NMNAT). NA is converted to NAMN by nicotinic acid phosphoribosyltransferase (NAPRT). NAMN is adenylated by NMNAT to nicotinic acid adenine dinucleotide (NAAD), which is amidated to NAD^+^ by NAD synthetase (NADS). Nucleosides NR and NAR are phosphorylated to NMN and NAMN, respectively, by the nicotinamide riboside kinases (NRK). NAD^+^ is cleaved to Nam during NAD^+^-dependent protein deacylation and ADP-ribosylation. NMN might also be synthesized by an extracellular NamPRT form (eNAMPT). NAD^+^ can possibly be released from cells through connexin 43 hemichannels (Cx43), and can be degraded to NR by ecto-nucleotidase CD73. NR is hydrolyzed to Nam by CD157. Extracellular NAD^+^ can also be hydrolyzed to Nam by CD38 and CD157.

The proper functional NAD-dependent regulation of metabolic and signaling processes depends on how efficiently cells can maintain a certain level of the dinucleotide. The major means to regulate cellular NAD levels is driven by the effective recycling of the endogenous precursor, nicotinamide (Nam), produced abundantly in deacylation and ADP-ribosylation processes. Another major means to maintain NAD levels is through its biosynthesis from extracellular precursors taken up from food sources: the vitamin B3 molecules which include Nam and nicotinic acid (NA) and their respective nucleosides, nicotinamide riboside (NR) and nicotinic acid riboside (NAR). The principal pathways of NAD^+^ biosynthesis in human cells have been well studied [4,5]. Following cellular uptake, the bases Nam and NA are converted to the corresponding mononucleotides, nicotinamide mononucleotide (NMN) and nicotinic acid mononucleotide (NAMN), by the phosphoribosyltransferases, nicotinamide phosphoribosyltransferase (NamPRT) and nicotinic acid phosphoribosyltransferase (NAPRT), respectively. Subsequently, the corresponding dinucleotides, NAD^+^ and NA adenine dinucleotide (NAAD) are formed by NMN adenylyltransferases (NMNATs) that add the adenylyl moiety of ATP-releasing inorganic pyrophosphate. In the two-step process from NAMN, NAAD is amidated to NAD^+^ by NAD synthase (NADS) (Figure 1). The nucleosides NR and NAR are also key metabolites for NAD^+^ biosynthesis in mammalian cells [6,7]. NR and NAR are phosphorylated to the corresponding mononucleotides by kinases of the nicotinamide riboside kinase (NRK) family. The subsequent conversions to generate NAD^+^ follow the same steps described above for Nam and NA (Figure 1). In an alternative route, the so-called de novo synthesis, tryptophan (Trp) is converted to quinolinic acid, which is a substrate of quinolinic acid phosphoribosyltransferase (QAPRT) that generates NAMN (Figure 1).

The mechanisms of NAD precursor uptake into human cells have so far not been sufficiently explored. It has been established that Trp uptake is mediated by the transporters SLC7A5 and SLC36A4, whereas NA is taken up through the carriers SLC5A8 and SLC22A13 [8,9,10,11]. The uptake routes for Nam, NR, and NAR remain unknown. Based on observations made using pharmacological inhibitors, we previously suggested that proteins belonging to the family of equilibrative nucleoside transporters (ENT) might mediate the transport of NR across the plasma membrane [12] (Figure 1).

Interestingly, exogenous nucleotides including NMN, NAMN, NAD^+^, and NAAD can support the maintenance of intracellular NAD pools as well as the nucleoside NR [12,13,14,15]. Moreover, several recent reports have shown that the application of NMN in various pathological animal models elevates tissue NAD levels and considerably improves physiological functions [16,17].

The mechanisms how the exogenous di- and mononucleotides, NAD^+^, NAAD, NMN, and NAMN, maintain intracellular NAD^+^ levels still needs clarification. It is known that extracellular NAD^+^ and NMN can be degraded to the corresponding nucleoside, NR, which thereafter enters the cell as precursor for NAD synthesis [12,18]. Moreover, the human ecto-enzyme CD73 has been described to catalyze both the cleavage of NAD^+^ to NMN and AMP as well as the subsequent dephosphorylation of the mononucleotides to the corresponding nucleosides, NR and adenosine [19,20]. In addition, extracellular NAD^+^ is used as a substrate of the glycohydrolases CD38 and CD157 which generate the second messengers ADP-ribose and cyclic ADP-ribose with the release of Nam [21] (Figure 1). Besides NAD^+^, the ecto-enzyme CD157 also efficiently hydrolyses NR, thereby generating Nam [22]. On the other hand, there are several studies supporting the direct uptake of NMN or NAD^+^ into human cells [13,23,24]. Moreover, recently the solute carrier SLC12A8 has been described as plasma membrane NMN carrier [25]. However, its physiological relevance has been questioned [26]. Clearly, there are still several open questions regarding precursor uptake and extracellular metabolism of NAD^+^ and its precursors.

In the present study, we applied a previously developed ^1^H-NMR-based metabolomics approach [27] to conduct a systematic analysis of the metabolism of exogenous NAD^+^ intermediates in cultures of human HEK293 cells and reveal how they are utilized to maintain intracellular NAD^+^ levels.

## 2. Results

### 2.1. Major Extracellular NAD Intermediates Support NAD Generation in HEK293 Cells

**Figure 2:**
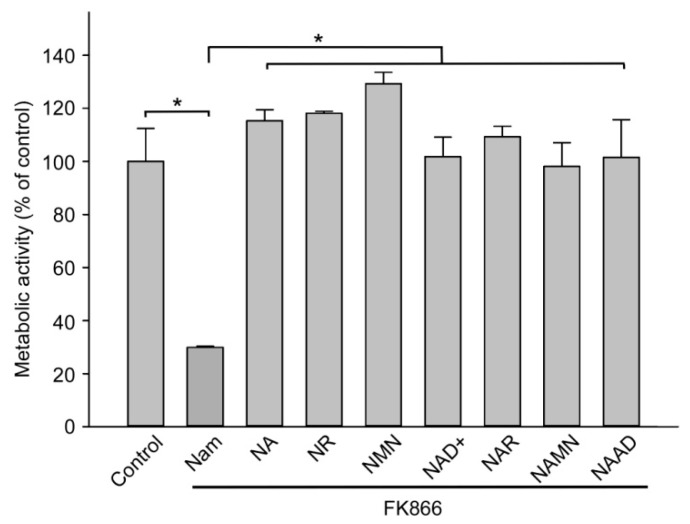
Extracellular NAD^+^ intermediates support NAD^+^ generation in HEK293 cells. HEK293 cells were cultivated in Dulbecco’s modified Eagle’s medium (DMEM) containing Nam, supplemented with 10% fetal bovine serum (FBS). To inhibit NAD^+^ synthesis from Nam, cells were treated with FK866. Cells were also treated with NAD^+^ or its derivatives as indicated. Metabolic activity was measured by MTT (3-(4,5-dimethylthiazol-2-yl)-2,5-diphenyltetrazolium bromide) assay 48 h after the treatment. Metabolic activity of untreated cells (control) was taken as 100%. Data are presented as mean ± S.D (*n* = 3). Statistical analysis of differences between the groups was carried out by one-way ANOVA with post hoc comparisons using Tukey test. * indicates statistical difference at *p* < 0.001.

In order to evaluate the capacity of extracellular NAD intermediates at maintaining intracellular NAD levels, we cultured HEK293 cells in standard culture medium (Dulbecco’s modified Eagle’s medium, DMEM) supplemented with 10% fetal bovine serum (FBS) which contains only nicotinamide as NAD precursor. Even though tryptophan is also known as NAD precursor, it is rarely used for NAD synthesis in these cells. Addition of the NamPRT inhibitor FK866 [28] to the medium leads to rapid depletion of the intracellular NAD stores and suppression of the metabolic activity of NAD(P)H-dependent dehydrogenases, which we estimated by MTT (3-(4,5-dimethylthiazol-2-yl)-2,5-diphenyltetrazolium bromide ) assay indirectly reflecting changes in NAD levels (Figure 2). When additionally supplemented with either the base NA, a nucleoside (NR or NAR), a mononucleotide (NMN or NAMN), or a dinucleotide (NAD^+^ or NAAD), the metabolic activity of the cells was restored to control levels (Figure 2). Consequently, all added major pyridine-containing NAD derivatives are efficient precursors of intracellular NAD.

### 2.2. Interconversion and Degradation of Extracellular NAD Intermediates in Cultures of HEK293 Cells

**Figure 3:**
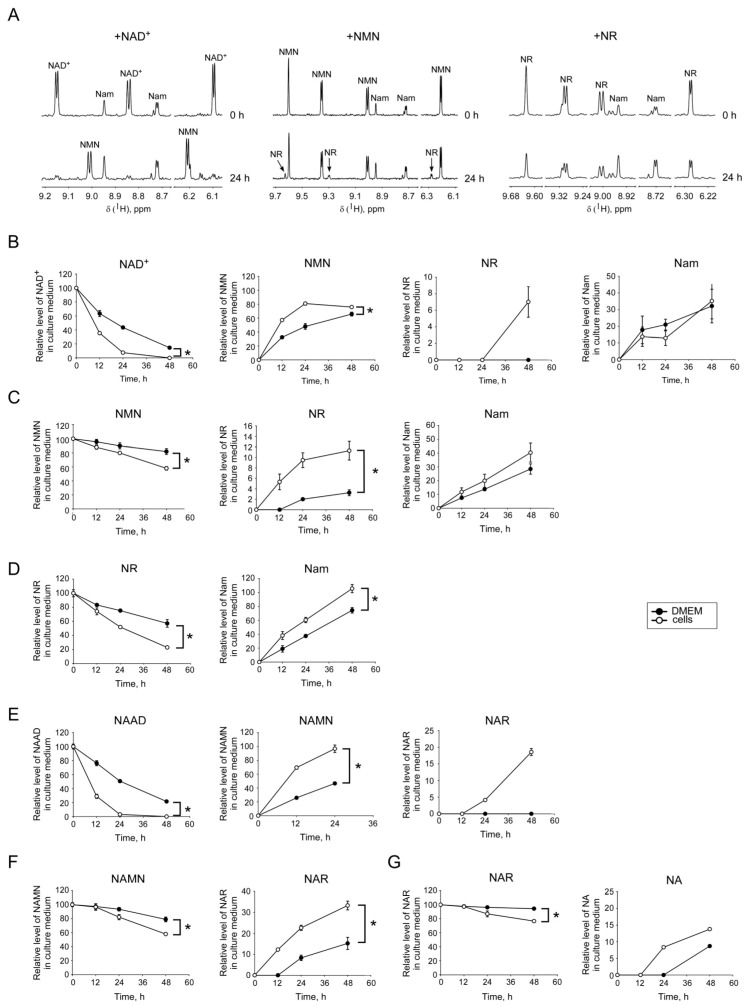
Degradation of extracellular NAD^+^ intermediates in cultures of HEK293 cells. (**A**) NAD+, NMN, or NR were added to the culture medium DMEM (containing Nam), supplemented with 10% FBS as indicated. Medium was then frozen (control, 0 h) or incubated with HEK293 cells for 24 h. ^1^H NMR spectra of control (0 h) and conditioned medium (24 h) are presented. NAD+ (**B**), NMN (**C**), NR (**D**), NAAD (**E**), NAMN (**F**), or NAR (**G**) were added to the culture medium DMEM (containing Nam), supplemented with 10% FBS. Medium was then frozen (control, 0 h) or incubated in the presence (white circles) or absence (black circles) of HEK293 cells for 12, 24 or 48 h and analyzed by NMR spectroscopy. (**B**–**G**) represent the relative levels of NAD intermediates added to the culture media (left panels) and their degradation products (other panels). Concentrations of metabolites added to culture media (0 h) were taken as 100. Concentrations of other metabolites were proportionally recalculated. Nam values are presented as estimated Nam amounts subtracted by Nam amount in a control (0 h). Data are presented as mean ± S.D (*n* = 3). Statistical analysis of differences between the groups was carried out by two-way ANOVA with post hoc comparisons using Tukey test. * indicates statistical difference at *p* < 0.001.

Next, we added a dinucleotide (NAD or NAAD), a nucleotide (NMN or NAMN) or a nucleoside (NR or NAR) at a concentration of 100 µM to the cells cultured in the same medium, but in the absence of FK866. Following incubation during various time intervals, we conducted a quantitative analysis of these compounds as well as their degradation products in the culture medium. The pyridine derivatives were measured using NMR spectroscopy. In Figure 3A, ^1^H NMR spectra are shown for the controls (t = 0 h) and after 24 h of incubation with either NAD^+^, NMN, or NR. After 24 h of incubation in the presence of cells, the amount of the added compounds was considerably lowered, while the accumulation of degradation products was observed. The quantitative analysis revealed that after 12 h of incubation in the presence of cells, less than 40% of the originally added amount of NAD^+^ remained. Moreover, after 48 h, NAD^+^ was undetectable in the medium (Figure 3B). At the same time, a considerable amount of NMN was detectable, but also the formation of Nam was observed. After 48 h, the medium contained another degradation product, namely, the nucleoside NR (Figure 3B). Surprisingly, NAD^+^ was efficiently degraded to NMN and Nam in the culture medium, even in the absence of cells. Within 24 h of incubation of NAD^+^ in the standard medium, DMEM supplemented with 10% heat-inactivated FBS; at 37 °C more than half of the added dinucleotide was degraded, whereas after 48 h less than 20% of the originally added amount remained (Figure 3B). The mononucleotide NMN exhibited a higher stability, even though it was also significantly degraded to NR and Nam in the absence of cells. In the presence of cells, NMN was degraded faster, but even under these conditions, after 24 and 48 h still 80% and 60%, respectively, of the added mononucleotide were present in the medium (Figure 3C). Likewise, we observed cleavage of NR to Nam in the medium without cells. However, when cells were present during the incubation, the level of NR was substantially lower, and that of Nam higher, compared to the incubation without cells (Figure 3D).

Similar experiments were performed with the deamidated NAD intermediates, NAAD, NAMN, and NAR. During incubation in the medium without cells, the dinucleotide NAAD was cleaved to the mononucleotide NAMN with similar efficiency as NAD was cleaved to NMN. NAMN, in turn, was cleaved to the corresponding nucleoside, NAR. In the presence of cells, the degradation of NAAD and NAMN was accelerated (Figure 3E,F). In contrast to the amidated pyridine nucleotides, NAAD and NAMN were not degraded to the base, NA. NAR was stable in the cell-free medium for 24 h. However, when cells were present, the level of NAR decreased by about 10% and the formation of NA became detectable (Figure 3G).

### 2.3. Fetal Bovine Serum (FBS) Has Pyridine Nucleotide-Degrading Activities

FBS, which is added to cell culture media, provides the cells with necessary hormones, growth factors, adhesion factors, and other proteins. To understand whether inactivated FBS contains enzymatic activities contributing to the observed degradation of NAD intermediates in the absence of cells, we incubated NAD^+^, NMN, or NR in DMEM supplemented with 10% FBS or with H_2_O for 24 h at 37 °C. Samples of the medium were then analyzed by NMR spectroscopy. Both NAD^+^ and NMN were relatively stable in control medium without FBS.

**Figure 4:**
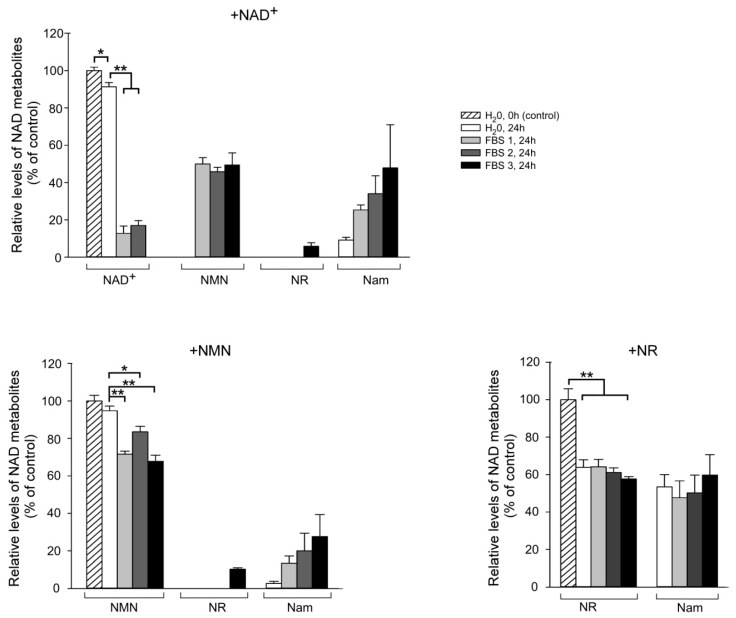
Degradation of NAD^+^, NMN and NR by FBS. NAD^+^, NMN, or NR were added to the culture medium DMEM (without Nam), supplemented with 10% FBS or H_2_O. FBS 1 was obtained from Gibco, FBS 2 was obtained from Biochrom, and FBS 3 was obtained from HyClone. Medium was then frozen (control) or incubated for 24 h at 37 °C. Relative levels of NAD^+^ intermediates in the samples were then estimated using quantitative NMR spectroscopy. Amounts of metabolites added to the control culture media were taken as 100%. Data are presented as mean ± S.D (*n* = 3). Statistical analysis of differences between the groups was carried out by one-way ANOVA with post hoc comparisons using Tukey test. * indicates statistical difference at *p* < 0.01, ** indicates statistical difference at *p* < 0.001.

Only about 10% or 5%, respectively, of the added metabolite underwent hydrolysis with the formation of Nam (Figure 4, upper and left lower panels). After addition of FBS, NAD^+^ and NMN degradation was substantial. FBS from three different manufacturers gave similar results. NAD^+^ was cleaved to NMN, while both NAD^+^ and NMN were degraded to Nam, only in one out of three cases to NR (Figure 4, upper and left lower panels). There are reports in the literature that even inactivated FBS may contain nucleotide pyrophosphatase and 5′-nucleotidase activities that degrade extracellular nucleotides such as ATP [29,30,31]. Obviously, pyridine mono- and dinucleotides also may be substrates of these enzymes. Our observations also suggest the presence of NADase activity in FBS as well as an enzyme that cleaves the Nam moiety from NMN.

Furthermore, we observed that during a 24 h incubation of NR in medium containing FBS from either of the three manufacturers about 35–40% of the nucleoside is cleaved to Nam (Figure 4, right lower panel). Strikingly, in the control sample that contained water instead of FBS, the extent of NR cleavage was similar (Figure 4, right lower panel). These data indicate that NR efficiently hydrolyses to Nam (and ribose) in aqueous solutions, while FBS does not seem to contain any detectable NR-degrading activity.

### 2.4. Cellular Ecto-Enzymes Metabolize Extracellular Pyridine Nucleotides

**Figure 5:**
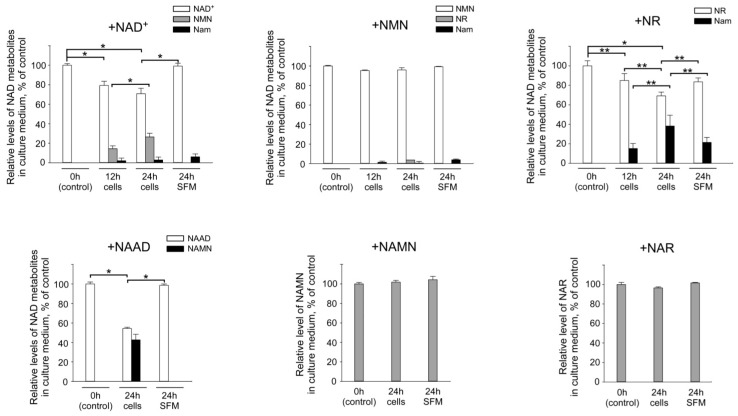
Degradation of extracellular NAD^+^ intermediates in cultures of HEK293 cells grown in serum-free medium. The indicated NAD^+^ intermediates were added to the serum-free medium (SFM) Pro293A-CDM. Medium was incubated in the presence or absence of HEK293 cells for 12, 24, or 48 h, as indicated. Relative levels of NAD^+^ intermediates in culture medium were then estimated using quantitative NMR spectroscopy. Amounts of metabolites added to culture media (control, 0 h) were taken as 100%. Nam values are presented as estimated Nam amounts subtracted by Nam amount in a control (0 h) medium. Data are presented as mean ± S.D (*n* = 3). Statistical analysis of differences between the groups was carried out by one-way ANOVA with post hoc comparisons using Tukey test. * indicates statistical difference at *p* < 0.001, ** indicates statistical difference at *p* < 0.05.

Next, we wished to clarify the role of cellular ecto-enzymes on the metabolism of extracellular pyridine derivatives. Therefore, we needed to exclude the contribution of the enzymes present in FBS. For this purpose, we conducted an adaptation of the HEK293 cells to FBS-free conditions in Pro293A-CDM medium. We then repeated the experiments described above with these cells in FBS-free medium and analyzed the degradation of added nucleotides. As shown in Figure 5, HEK293 cells efficiently metabolize both NAD^+^ and NAAD, cleaving them to the corresponding mononucleotides. Interestingly, the levels of NMN and NAMN did practically not change upon incubation with cells in FBS-free medium. During 24 h of incubation of cells with NMN, only about 5% of the mononucleotides were converted to NR, whereas no degradation products of NAMN (NAR and NA) were detectable (Figure 5). NR degradation was similar in the Pro293A-CDM medium to that observed in the FBS-supplemented medium without cells present. However, when cells were present, NR conversion to Nam was substantially enhanced (Figure 5). NAR, however, was rather stable irrespective of the presence of cells.

These results suggest that exogenous dinucleotides are cleaved by cellular ecto-enzymes to the corresponding mononucleotides. In the absence of FBS, NMN is far more stable and only rather slowly dephosphorylated to NR. NR, in turn, is actively cleaved to form Nam. Interestingly, the degradation of NMN and, in particular, NR appears to be specific for the amidated intermediates, as no cleavage of NAMN or NAR was detected. It should be noted that the results presented above do not formally rule out the possibility that NAD+ and its intermediates are imported to cells and then cleaved by intracellular enzymes.

### 2.5. ENT Inhibition Suppresses NR, NMN, and NAD^+^ Utilization by HEK293 Cells

**Figure 6:**
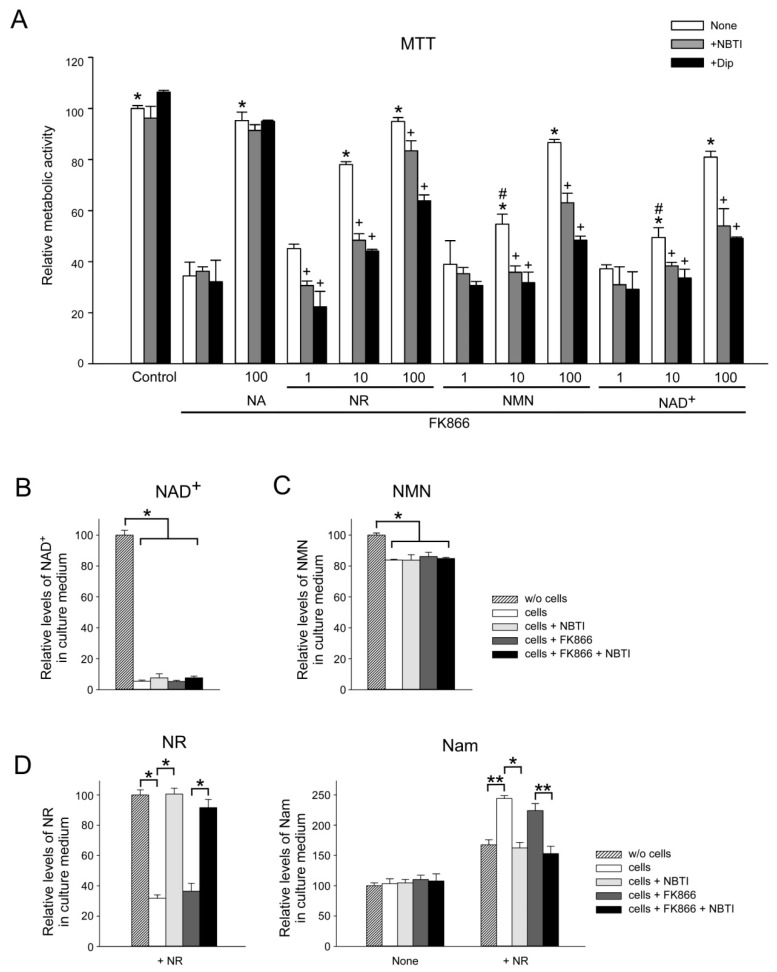
The effect of equilibrative nucleoside transporter (ENT) inhibition on NR, NMN, and NAD^+^ utilization by HEK293 cells. HEK293 cells were cultivated in the serum-free medium Pro293A-CDM in the presence of NA, NR, NMN, or NAD^+^ at a concentration of 1 µM, 10 µM, or 100 µM, as indicated (**A**) or in the presence of NAD^+^ (**B**), NMN (**C**) or NR (**D**) at a concentration of 100 µM. Cells were also treated with inhibitors of equilibrative nucleoside transporters S-(4-nitrobenzyl)-6-thioinosine (NBTI) or dipyridamole (Dip). Nam utilization was inhibited by FK866 addition. (**A**) Metabolic activity was measured by MTT assay 72 h after the treatment. Metabolic activity of untreated cells was taken as 100. (**B**) Relative levels of NAD metabolites in culture media were estimated using quantitative NMR spectroscopy 48 h after the treatment. Amounts of NAD^+^, NMN, NR, and Nam in control culture media incubated without cells were taken as 100. Data are presented as mean ± S.D (*n* = 3). Statistical analysis of differences between the groups was carried out by two-way ANOVA with post hoc comparisons using Tukey test. * indicates statistical difference at *p* < 0.001 vs. the FK866-treated cells, # indicates statistical difference at *p* < 0.001 vs. treatment with the 10 µM NR, + indicates statistical difference at *p* < 0.01 vs. the treatment with corresponding NAD precursor only. (**B**–**D**) Relative levels of NAD metabolites in culture media were estimated using quantitative NMR spectroscopy 48 h after the treatment. Concentrations of NAD^+^, NMN, NR, and Nam in control culture media incubated without cells (*w*/*o* cells) were taken as 100. Data are presented as mean ± S.D (*n* = 3). Statistical analysis of differences between the groups was carried out by one-way ANOVA with post hoc comparisons using Tukey test. * indicates statistical difference at *p* < 0.001, ** indicates statistical difference at *p* < 0.01.

The results indicated that at least the nucleosides are direct precursors of intracellular NAD biosynthesis. Therefore, we next studied to what extent the inhibition of plasma membrane nucleoside transporters of the ENT family would affect the maintenance of intracellular NAD levels using different types of extracellular NAD^+^ metabolic intermediates. For this purpose, we cultured HEK293 cells in FBS-free medium (Pro293A-CDM) in the presence of FK866. We also added to the medium a NAD^+^ precursor (NAD^+^, NMN, NR, or NA) in different concentrations (1, 10, or 100 µM) as well as nucleoside transport inhibitors, S-(4-nitrobenzyl)-6-thioinosine (NBTI) or dipyridamole (Dip). As shown in Figure 6A, NA completely restored the NAD-dependent metabolic activity of cells treated with FK866. The inhibitors NBTI and Dip did not influence the efficiency of NA to maintain NAD-dependent activities. Low concentrations (1 µM) of NR, NMN, or NAD^+^ did not efficiently restore intracellular NAD pools. It was found that 10 µM of NR, but not NMN or NAD^+^, restored the metabolic activity of the cells to about 80% of control cells. NR, NMN, and NAD^+^ were efficient at 100 µM. However, both inhibitors, NBTI and Dip, suppressed the restoration of intracellular NAD pools by these intermediates (Figure 6A). It was also established that neither the inhibition of the nucleoside transport by NBTI nor the inhibition of NamPRT by FK866 had any influence on the extracellular cleavage of NMN and NAD^+^ (Figure 6B,C). These data indicate that NR represents the most efficient amidated extracellular precursor of intracellular NAD. Moreover, at least in HEK293 cells, the extracellular cleavage of NAD^+^ and NMN to NR appears to be a prerequisite for their effectiveness as extracellular precursors.

Inhibition of NAD^+^ synthesis from Nam by FK866 did also not influence the extent of NR utilization (Figure 6D, left panel). On the other hand, the efficient utilization of NR and concomitant Nam formation was completely blocked by NBTI (Figure 6D). These data suggest that NR is not cleaved outside the cells, but rather is taken up into the cells by a NBTI-sensitive carrier to be efficiently metabolized into NAD which is then converted to Nam through NAD-consuming processes (Figure 1). Alternatively, intracellularized NR or its phosphorylated form could be converted to Nam prior to its conversion to NAD by glycolytic enzymes. On both accounts, Nam is then released from the cells into the culture medium where it is detected.

## 3. Discussion

The present study has demonstrated that all known intermediates of NAD biosynthesis, including NAD^+^ itself, can serve as extracellular precursors of intracellular NAD. However, there is an active conversion of pyridine nucleotides and nucleosides in the medium of cultured HEK293 cells, and our results support the conclusion that the extracellular degradation of the mono- and dinucleotides is a prerequisite for their suitability to maintain intracellular NAD levels. We were able to separate the contributions of enzymatic activities originating from the culture medium, especially the FBS, and the cells themselves by conditioning the cells to a medium not containing FBS.

Because of various beneficial effects, there is currently a great interest in the possibilities to maintain or even enhance intracellular NAD levels [16,17]. As shown here, any intermediate of NAD metabolism could potentially contribute to the maintenance of intracellular NAD. However, it is unlikely that they are equally relevant for this function. Physiologically, the intermediates are differently available. The bases Nam and NA are well-described vitamins that are present in many food sources. Moreover, Nam is also the by-product of all known NAD-dependent signaling reactions. Once produced in the cells, Nam can be recycled into NAD or exit the cells into the extracellular space. In recent years, the attention was also paid to the fact that there are other nutritional NAD precursors such as NR and NMN [32,33].

In addition, the secretion of NamPRT from cells has been described, which, in principle, would enable the generation of NMN in the extracellular environment [34,35]. The sources and necessity of extracellular NAD^+^ have not been firmly established. However, extracellular NAD^+^ levels (for example in blood plasma) have been measured under various conditions with values ranging from submicromolar to up to 10 µM [36,37,38]. Extracellular NAD^+^ could be the result of cell lysis. It has also been reported that NAD^+^ can be exported from cells through connexin 43 hemichannels [39,40]. Moreover, stressed cells can actively release NAD^+^. In fact, it has been proposed that NAD^+^ could serve as neurotransmitter or neuromodulator [41,42,43].

With the exception of NA, the presence of the deamidated pyridine nucleotide derivatives in extracellular fluids has hardly been investigated so far. We have previously shown that human cells can synthesize and release NAR, which thereafter can be utilized by other cells as NAD precursor [7].

The detection of di- and mononucleotide-degrading activities in FBS was not surprising. There have been reports describing the presence of nucleotide pyrophosphatase and 5′-nucleotidase activities, even in inactivated FBS. These enzymes cleave extracellular nucleotides such as ATP [29,30,31] and may well be those detected here to cleave pyridine nucleotides. Our results furthermore indicate the presence of a NADase activity which could possibly also take NMN as a substrate, because Nam was identified as a degradation product of both nucleotides. Interestingly, NR tends to hydrolyze far more readily than the other molecules studied, however, there is no detectable NR degrading activity in FBS.

The exogenous dinucleotides NAD^+^ and NAAD are cleaved by the cultured human cells to the corresponding mononucleotides, NMN and NAMN. NMN exhibits a relatively high chemical stability but is partly dephosphorylated to NR by the cells. These data are in line with previous observations in HepG2 cells grown in serum- and nicotinamide-free medium [18]. On the other hand, the degradation of NAMN and NAR is significantly slower compared to their amidated counterparts. This is an intriguing observation, since it indicates that the enzymes involved exhibit a substrate selectivity with a preference for the amidated pyridines.

NR added to the cell culture is efficiently cleaved to Nam. Our experiments have shown that this conversion occurs following uptake of NR mediated by NBTI-sensitive channels inside the cells. The produced Nam is then released back into the medium where its accumulation is detectable. Interestingly, in HepG2 cells, over time, a considerable decrease of added NR was observed, while no accumulation of Nam was detected [18]. These observations may indicate slight differences in the NAD metabolism of the cell lines used.

The experiments using inhibitors of plasma membrane nucleoside transporters (NBTI, Dip) have also reinforced the notion that, at least in HEK293 cells, the degradation of NAD^+^ and NMN to NR or Nam is essential for these nucleotides to act as extracellular precursors of intracellular NAD. Of course, we cannot exclude off-target effects of the inhibitors. However, they have been comprehensively characterized as highly selective.

In conclusion, our analyses of extracellular conversions of vitamin B3-derived NAD^+^ derivatives have shown an active role of both the culture medium and the cells themselves. While deamidated molecules (e.g., NAR, NAMN) appear chemically more stable than NR and NMN, their cellular utilization is much slower. The results of this study imply that the culture conditions can have a profound impact on the chemical and enzymatic stability of NAD precursors added to the medium. This needs to be taken into account when studying precursor uptake and conversions.

## 4. Materials and Methods

### 4.1. Materials

Unless otherwise specified, all chemicals and reagents were of analytical grade and were purchased from Sigma (Saint Louis, MO, USA) and Amresco. Cell culture reagents were from Gibco (Waltham, MA, USA), HyClone (Pittsburgh, PA, USA), Biochrom (Berlin, Germany), Greiner Bio-One (Monroe, NC, USA), and Orange Scientific (Braine-l’Alleud, Belgium). HPLC-grade acetonitrile was obtained from Merck (Darmstadt, Germany). The ultrapure water was obtained from a Milli-Q Synthesis purification system (Millipore, Burlington, MA, USA). NR and NAR were synthesized as reported previously [44].

### 4.2. Cell Culture

HEK293 cells (obtained from American Type Culture Collection (ATCC)) were cultivated in Dulbecco’s modified Eagle’s medium (DMEM) or serum free medium Pro293A-CDM (Lonza). Media were supplemented with 10% fetal bovine serum (FBS), 2 mM glutamine, and penicillin/streptomycin. The cells were cultured at 37 °C in a humidified atmosphere of 5% CO_2_. Metabolic activity was determined using 3-(4,5-dimethylthiazol-2-yl)-2,5-diphenyltetrazolium bromide (MTT) assay.

### 4.3. Pharmacological Treatments

Unless otherwise specified, NA, NR, NAR, NMN, NAMN, NAD^+^, and NAAD were added to the cell culture medium at a concentration of 100 µM. Cells were also treated with inhibitors: S-(4-nitrobenzyl)-6-thioinosine (NBTI) at a concentration of 10 µM, dipyridamole at a concentration of 2 µM, and FK866 at a concentration of 2 µM. Treatment with metabolites and inhibitors was carried out for 12–72 h prior to NMR analyses or MTT assay.

### 4.4. NMR Sample Preparation

Culture media were collected and stored at −80 °C. To precipitate proteins, the samples were incubated on ice with 2 volumes of acetonitrile for 30 min and then centrifuged at 15,000× *g* for 30 min at 4 °C. Supernatants were lyophilized and resuspended in D_2_O-based buffer containing 50 mM NaPi (pH 6.5) and 1 mM sucrose as a chemical shift reference (δ(^1^H), 5.42 ppm) and internal standard for quantification. Samples were stored at −80 °C until NMR analysis.

### 4.5. NMR Analysis

All NMR experiments were performed using a Varian DirectDrive NMR System 700-MHz spectrometer equipped with a 5-mm z-gradient salt-tolerant as described in [27]. Briefly, the one-pulse sequence with the suppression of solvent signal by presaturation was used for acquisition of ^1^H spectra. The following acquisition parameters were used: relaxation delay, 2.0 s; acquisition time, 3.0 s; and number of scans, 128–8192. Data were acquired using VNMRJ 4.2 (Agilent Technologies, Santa Clara, CA, USA) and then analyzed by Mestrelab Mnova (version 12; Mestrelab, Santiago de Compostela, Spain). The concentrations of metabolites were determined by integration of the corresponding non overlapping proton signals.

### 4.6. Statistical Analysis

Statistical analysis was performed using the SigmaPlot 12.0 (Systat Software Inc., San Jose, CA USA). Differences between groups were analyzed using one-way or two-way ANOVA with Tukey’s post-hoc test as indicated in the figures. *p*-values <0.05 were considered to be significant.
